# Sentinel Lymph Node Detection in Contralateral Axilla at Initial Presentation of a Breast Cancer Patient: Case Report

**DOI:** 10.4274/mirt.91300

**Published:** 2015-06-17

**Authors:** Gülin Uçmak Vural, Ilgın Şahiner, Semra Demirtaş, Hülya Efetürk, Bedriye Büşra Demirel

**Affiliations:** 1 Ankara Oncology Hospital, Clinic of Nuclear Medicine, Ankara, Turkey

**Keywords:** sentinel lymph node, lymphoscintigraphy, contralateral axilla, Breast cancer

## Abstract

The main basin for breast lymphatic drainage is ipsilateral axilla. However, extra-axillary drainage may be seen in some patients. The most common extra-axillary site is internal mammary chain, while contralateral axillary drainage is an extremely rare situation in previously untreated patients. We describe a case of untreated right breast retroareolar carcinoma with contralateral axillary drainage detected on preoperative lymphoscintigraphy. Contralateral axillary dissection was performed based on the result of frozen section examination of the sentinel lymph node (SLN) which turned out to burden micrometastasis. Postoperative histopathological examination revealed invasive ductal carcinoma metastasis in 17 out of 22 lymph nodes from the ipsilateral axillary dissection, whereas 14 lymph nodes from contralateral axillary dissection other than the SLN were nonmetastatic. In our opinion, determination of contralateral axillary metastasis in primary staging process had a major contribution to the management of the patient.

## Abstract (tr)

Memenin lenfatik drenajı ilk olarak ipsilateral aksillaya olmakla birlikte, bazı hastalarda aksilla-dışı drenaj paterni izlenebilmektedir. En sık izlenen aksilla-dışı odak internal mammarian zincir olup, karşı aksillaya drenaj, önceden cerrahi girişim geçirmemiş hastalarda, çok nadir gözlenen bir durumdur. Bu çalışmada yeni tanı sağ meme retroareolar karsinomlu hastada, preoperatif dönemde yapılan lenfosintigrafide kontralateral aksillaya drenaj sunulmaktadır. Hastaya, cerrahi sırasında karşı taraf sentinel lenf nodunda frozen incelemesi sonucu mikrometastaz tespit edilmesi nedeniyle, kontralateral aksiller diseksiyon yapılmıştır. Postoperatif histopatolojik incelemeler ipsilateral aksiller diseksiyonda çıkarılan 22 lenf nodunun 17’sinde invaziv duktal karsinom metastazını göstermiştir. Kontralateral aksillada ise, sentinel lenf nodu dışında çıkarılan 14 lenf nodunun hiçbirinde metastaz saptanmamıştır. Bu olguda, primer evreleme sürecinde karşı taraf aksillaya olan metastazın saptanmasının hastanın tedavi planına büyük katkı sağladığını düşünmekteyiz.

## INTRODUCTION

The main basin for breast lymphatic drainage is ipsilateral axilla. However, prior surgery and radiotherapy to the breast or axilla, and malignant infiltration of lymphatics and lymph nodes may result in aberrant routes of drainage (1). Extra-axillary drainage of the breast is mainly to the internal mammary chain, and to a lesser extent to intramammary, subclavicular, interpectoral and supraclavicular lymph nodes (2). Although extra-axillary drainage may be expected in patients with previously treated breast cancer, this situation is uncommon in untreated patients (2,3,4). Contralateral axilla is a relatively rare basin for lymphatic drainage of breast tumors. It is postulated that lymphatic blockage by tumoral infiltration or damage caused by surgical intervention might lead to formation of alternative routes of lymphatic drainage.

In clinically node negative patients with breast cancer, sentinel lymph node biopsy is recommended for axillary staging (5). Lymphatic mapping with lymphoscintigraphy followed by radioguided detection of the sentinel lymph node using gamma probe is a well-established technique with high identification rates (6).

We describe a case of previously untreated right breast retroareolar carcinoma with contralateral axillary drainage detected on preoperative lymphoscintigraphy on the day of surgery.

## CASE REPORT

A 34-year-old female presented with complaints of right nipple retraction and right breast pain. The physical examination of the right breast revealed a retroareolar tumor together with periareolar edema of the skin, and nipple retraction. Physical examination of the right axilla was normal. A retroareolar tumor with ill-defined contours measuring 2 cm with calcifications was detected on mammography (BIRADS 4C). Physical examination and mammography of the left breast and axilla were normal.

The excisional biopsy identified a grade 3 invasive ductal carcinoma. The invasive tumor measuring 1.5 cm in largest diameter, was positive for estrogen (70%) and progesterone (70%) receptors, and negative for cerbB2. Ki-67 index was 40%. Preoperative chest X-ray, abdominal ultrasound and bone scintigraphy were normal. Serum carcinoembyronic antigen (CEA) level was 2.01 ng/mL (normal range: 0-4 ng/mL) and cancer antigen 15-3 (CA 15-3) level was 34.6 U/mL (normal range: 0-38.6 U/mL).

**Figure 1:**
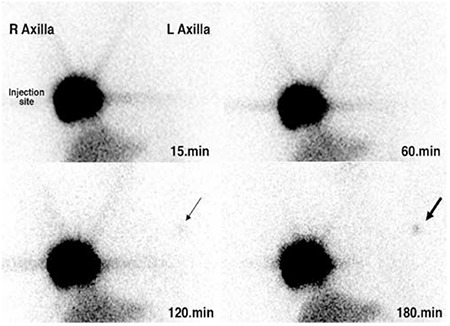
Preoperative lymphoscintigraphy, demonstrating lack of ipsilateral lymphatic drainage in the right axilla up to 180 minutes after injection time. Serial static imaging demonstrated contralateral axillary sentinel lymph node as faint focal uptake in the left axillary region 120 minutes after injection (thin arrow, lower left row), and a more prominent focal uptake (thick arrow, lower right row) 180 minutes after application of radioactivity decontamination solution

Preoperative lymphatic mapping was performed on the day of the operation. Intradermal injections Tc-99m albumin colloid (NanoCIS; Cis Bio International) each containing activities of 0.1 mCi were applied periareolarly and intraparenchymally to the biopsy site. No ipsilateral axillary drainage was seen on lymphoscintigraphy, whereas focal radiocolloid uptake was detected in the contralateral axilla (Figure 1).

The sentinel lymph node in the contralateral axilla was detected by using gamma probe during surgery. Frozen section examination of the material identified breast cancer micrometastasis with a maximal diameter of 0.5 mm. Modified radical mastectomy of the right breast, left and right axillary dissection (level I/II) were performed. Postoperative histological examination revealed invasive ductal carcinoma metastasis in 17 out of 22 lymph nodes from the right axillary dissection, whereas 14 lymph nodes from left axillary dissection other than the SLN were nonmetastatic.

The patient was regarded as having stage IIIC disease, adjuvant chemotherapy including docetaxel, doxorubicin and cyclophosphamide was started 5 weeks after surgery, and postoperative external beam radiotherapy for ipsilateral chest wall was scheduled.

## LITERATURE REVIEW AND DISCUSSION

Lymphatic drainage to contralateral axilla and to both axillae, in the absence of ipsilateral metastatic lymph nodes has previously been reported in a few cases (7,8). Although non-visualization of sentinel lymph node on lymphoscintigraphy as well as extra-axillary drainage in case of tumor infiltration of lymphatics and lymph nodes are expected, to our knowledge contralateral SLN detection in a patient with ipsilateral positive lymph nodes has only been reported once (9). In the case previously reported by Stevens H. et al. lymphoscintigraphy demonstrated a contralateral axillary SLN in a lateral mid quadrant right breast tumor which turned out to be ductal carcinoma with a diameter of 4.3 cm on histopathological examination. Dissection of ipsilateral axillary nodes and four nodes from the contralateral axilla revealed metastases in all ipsilateral and two contralateral nodes. However, the reason for not performing dissection to the contralateral axilla was not reported. Contrary to the previous report, SLN in the contralateral axilla detected in our patient was the only micrometastatic focus along with all ipsilateral axillary metastatic nodes.

The patient was considered to be node positive rather than having metastatic disease since the preoperative lymphoscintigraphy demonstrated contralateral lymphatic drainage. Detection of micrometastatic disease in the contralateral axilla would have been impossible if the preoperative scan was not carried out in the present case, and the patient would probably present with contralateral axillary metastasis (CAM) upstaging the case in the future. When malignancy is detected in the contralateral axillary lymph nodes, extensive work up of the breast and whole body is warranted in order to exclude the presence of ipsilateral occult foci as well as metastatic spread of an extramammarian malignancy. Presence of CAM is regarded as distant metastasis, since contralateral axilla is not a regional draining basin of the breast. There is controversy in the management of CAM, especially when metastatic disease is not present elsewhere. From this point of view, we believe that preoperative detection of contralateral drainage route by lymphoscintigraphy facilitated patient management by individualizing therapy plan in this rare situation.

If radioactive colloid is to be used for SLN identification, whether routine preoperative lymphoscintigraphy should be used or not is controversial due to the additional time required to perform the method and increased costs related to its application (6,10). Some centers eliminate the imaging procedure, because lymphatic drainage of the breast is mostly predictable, with the majority of lesions draining to axillary nodes. However, lymphoscintigraphy is the only method to detect extra-axillary drainage sites. Moreover, it aids surgeons especially in cases where intraoperative detection rates are low due to patient related factors such as obesity and advanced age (6). In addition, lymphoscintigraphy also serves to predict the patients in which the sentinel lymph node biopsy procedure will fail during surgery. Using only the blue dye technique, this situation will not be evident preoperatively.

In the present case, lymphatic drainage was detected in the contralateral axilla during late stages of the scan. This situation raised suspicion of ipsilateral axillary involvement. This case highlights the importance of sequential imaging with preoperative lymphoscintigraphy for detection of SLN in addition to usage of intraoperative gamma probe. Although gamma probe detection of SLN has higher sensitivity rate as compared to imaging with gamma camera, in order to obtain better guidance scanning procedure should not be omitted. It should also be kept in mind that delayed images detect more nodes than early imaging (11,12). Furthermore, non-visualization of SLN in lymphoscintigraphy is associated with the presence of more metastatic lymph nodes (12). These issues must be closely considered especially in circumstances where the axillary nodes are clinically suspicious.

Sentinel node biopsy is not recommended in patients with clinically positive axilla due to high rates of false negativity and high risk of procedural failure in identifying the SLN (13,14). However, unexpected draining nodes are more common in patients with nodal metastasis, due to obstruction of lymphatic vessels. In the case presented herein, lymph nodes of the ipsilateral axilla turned out to be metastatic although they were clinically non-palpable. In our opinion, late visualization of the contralateral metastatic lymph node resulted from ipsilateral blockage of lymphatics.

Preoperative lymphoscintigraphy is a valuable tool in radioguided detection of sentinel lymph nodes. We believe that further clinical studies should be conducted in order to evaluate the role of lymphoscintigraphy for detection of extra-axillary sentinel lymph nodes in patients with clinically node positive breast cancer in whom axillary lymph node dissection is already planned.
